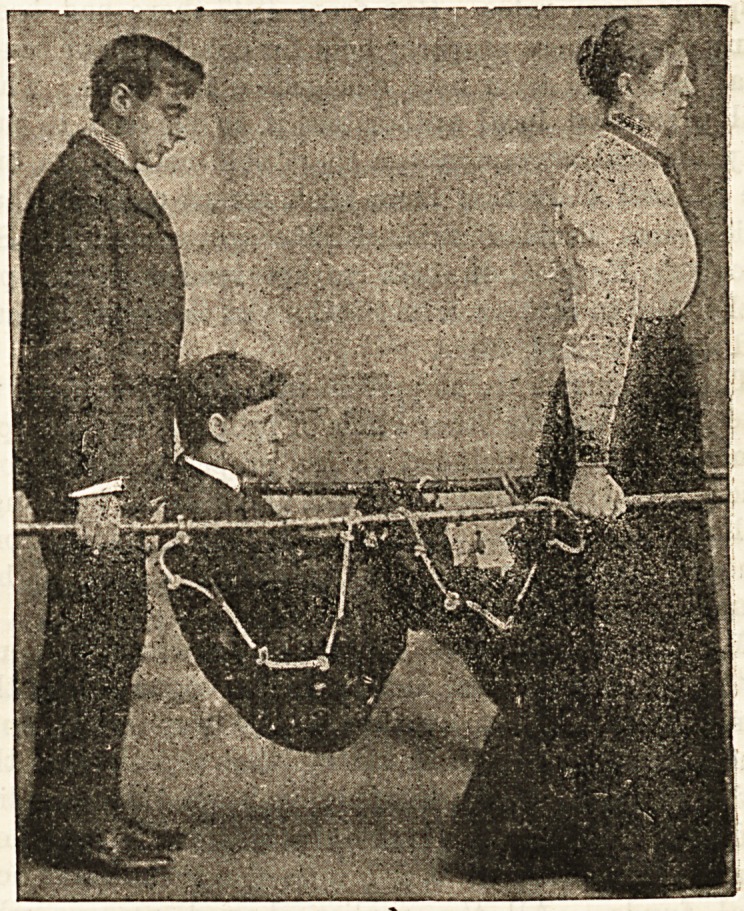# New Appliances and Things Medical

**Published:** 1900-08-18

**Authors:** 


### NEW APPLIANCES AND THINGS MEDICAL.

[We shall be glad to receive, at our Office, 28 & 29, Southampton Street, Strand, London, W.C., from the manufacturers, specimens of all new preparations and appliances which may be brought out from time to time.]

### CARRYING SHEET STRETCHER.

(Messrs. W. H. Bailey and Son, 38, Oxford Street, London, W.)

Mr. J. C. Derham, the chief constable of Blackpool, has devised a useful form of carrying sheet, which has been adapted by Mrs. Alfred Paine, of Bedford, as a stretcher, the result being a very handy means of transport for the sick or injured. The trick about the carrying sheet is that all along the sides it has a series of brass eyelet holes, through which is passed a soft and strong cotton rope, knotted at certain distances in such a way as to form handles or loops that can be easily grasped. Anyone who has ever helped to carry a patient in a blanket will recognise at once what an advantage it is to be able to "catch hold of" something firm at any required point. The transformation of this sheet into a stretcher is a simple affair, and is soon done by slipping a couple of carrying poles through the loops of the rope, and keeping them apart if required by the insertion of a transverse bar at one or at both ends. The great advantage which this stretcher offers over the ordinary form in which the carrying poles run in the hem is that, by missing a loop here and there in threading the poles, one can make the stretcher fit much more nearly to the contour of the body than one could if every part of it were equally supported. The accompanying figures show at once how adaptable the contrivance is. It can be used without the poles as a carrying sheet for stairs and awkward corners, or with them as an ordinary stretcher, as a carrying chair, as a bed lift, or as a hammock, and it offers great facilities for railway or omnibus transport in consequence of the facility with which it can be narrowed by the temporary removal of the cross pieces for the sake of getting through the doorways.

## Figures and Tables

**Figure f1:**
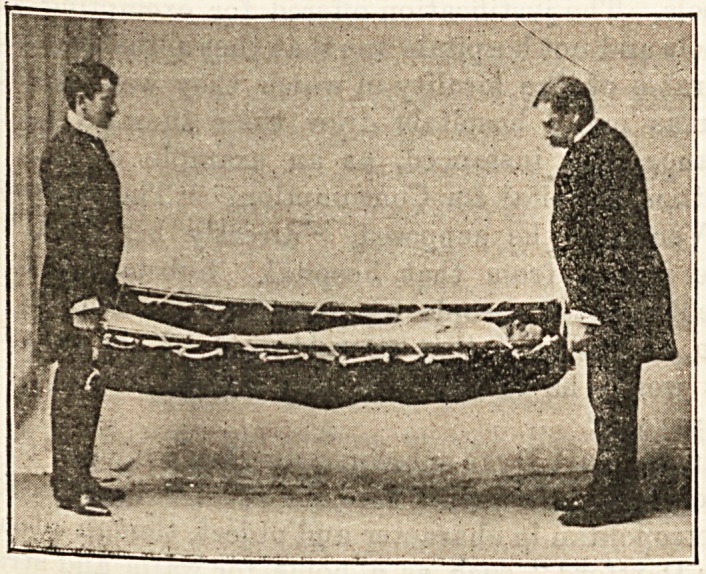


**Figure f2:**